# Historical isolation and contemporary gene flow drive population diversity of the brown alga *Sargassum thunbergii* along the coast of China

**DOI:** 10.1186/s12862-017-1089-6

**Published:** 2017-12-07

**Authors:** Jing-Jing Li, Zi-Min Hu, Zhong-Min Sun, Jian-Ting Yao, Fu-Li Liu, Pablo Fresia, De-Lin Duan

**Affiliations:** 1 Key Laboratory of Experimental Marine Biology, Institute of Oceanology, Chinese Academy of Sciences, Qingdao, 266071 China; 2 Laboratory for Marine Biology and Biotechnology, Qingdao National Laboratory for Marine Science and Technology, Qingdao, 266071 China; 3 Institute of Marine Biology, College of Oceanography, Hohai University, Nanjing, 210098 China; 4 Laboratory of Marine Organism Taxonomy & Phylogeny, Institute of Oceanology, Chinese Academy of Sciences, Qingdao, 266071 China; 5 Key Laboratory of Sustainable Development of Marine Fisheries, Ministry of Agriculture, Yellow Sea Fisheries Research Institute, Chinese Academy of Fishery Sciences, Qingdao, 266071 China; 6 Unidad de Bioinform atica, Institut Pasteur de Montevideo, Mataojo, 2020 Montevideo, Uruguay

**Keywords:** Gene flow, Historical isolation, Long-distance dispersal, Microsatellite, Plastid RuBisCo spacer, Population genetic diversity, *Sargassum thunbergii*

## Abstract

**Background:**

Long-term survival in isolated marginal seas of the China coast during the late Pleistocene ice ages is widely believed to be an important historical factor contributing to population genetic structure in coastal marine species. Whether or not contemporary factors (e.g. long-distance dispersal via coastal currents) continue to shape diversity gradients in marine organisms with high dispersal capability remains poorly understood. Our aim was to explore how historical and contemporary factors influenced the genetic diversity and distribution of the brown alga *Sargassum thunbergii*, which can drift on surface water, leading to long-distance dispersal.

**Results:**

We used 11 microsatellites and the plastid RuBisCo spacer to evaluate the genetic diversity of 22 *Sargassum thunbergii* populations sampled along the China coast. Population structure and differentiation was inferred based on genotype clustering and pairwise *F*_ST_ and allele-frequency analyses. Integrated genetic analyses revealed two genetic clusters in *S. thunbergii* that dominated in the Yellow-Bohai Sea (YBS) and East China Sea (ECS) respectively. Higher levels of genetic diversity and variation were detected among populations in the YBS than in the ECS. Bayesian coalescent theory was used to estimate contemporary and historical gene flow. High levels of contemporary gene flow were detected from the YBS (north) to the ECS (south), whereas low levels of historical gene flow occurred between the two regions.

**Conclusions:**

Our results suggest that the deep genetic divergence in *S. thunbergii* along the China coast may result from long-term geographic isolation during glacial periods. The dispersal of *S. thunbergii* driven by coastal currents may facilitate the admixture between southern and northern regimes. Our findings exemplify how both historical and contemporary forces are needed to understand phylogeographical patterns in coastal marine species with long-distance dispersal.

**Electronic supplementary material:**

The online version of this article (10.1186/s12862-017-1089-6) contains supplementary material, which is available to authorized users.

## Background

**Fig. 1:**
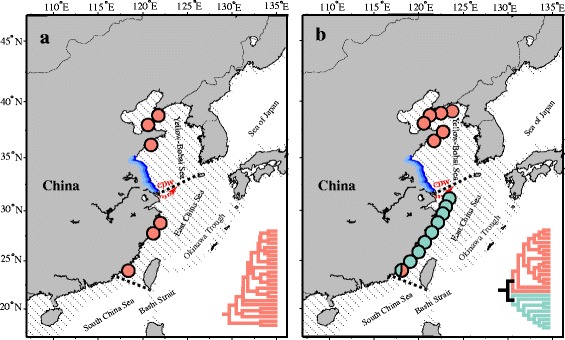
Two biogeographic patterns for intertidal species in Yellow-Bohai Sea and East China Sea. Lineage distribution and neighbor-joining tree were redrawn from (**a**) *Sargassum horneri* [4] and (**b**) *Sargassum fusiforme* [16]. Shaded sea areas are continental shelves that would have been exposed to the air during periods of low sea level. CDW: Changjiang diluted water. Blue line: the coastline of Jiangsu Province

The past decade has witnessed extensive research regarding genetic diversity and the evolutionary history of marine species on the coast of China [1–5]. This is due, in part, to the fact that the coast of China is characterized by distinct tectonic and geological processes, with a series of marginal seas separating the eastern Asian continent from the Pacific Ocean [6]. During the Last Glacial Maximum (LGM, 0.026–0.019 Ma), the drastic decline of sea levels (*c*. 135 m lower than today) restructured the marginal seas in the Northwest Pacific; the beds of the Yellow-Bohai Sea (YBS) were entirely exposed and the East China Sea (ECS) was reduced to an elongated trough (the Okinawa Trough). The ECS and South China Sea (SCS) were thus isolated due to a land bridge extending from eastern China to Taiwan Island. The Sea of Japan (SOJ) also became an enclosed inland sea [6–8] (Fig. 1). Such restructuring of the marginal seas isolated populations of coastal marine species along the coast of China, resulting in dynamic phylogeographical signatures in present populations [5, 9, 10]. Worldwide, in marine environments, historical glaciation is proved to be the most effective force in generating intraspecific genetic splits [11], such as in Indo-West Pacific [12], South East Pacific [13] as well as North East Atlantic [14]. Two biogeographic scenarios have been proposed for species isolated in the YBS/ECS basin (Fig. 1): I) species possessed a homogeneous population structure as reported in the barnacle *Chthamalus challengeri* [3] and the alga *Sargassum horneri* [4] (Fig. 1a); and II) species comprised two genetic clusters (YBS vs. ECS) which were separated by the line of Changjiang estuary, as illustrated in the gastropod *Cellana toreuma* [15] and the brown alga *Sargassum fusiforme* [16] (Fig. 1b). These contrasting biogeographic patterns indicate that coastal species co-distributed along the coast of China might exhibit different ecological responses to climatic shifts.

Gene flow is another important factor driving biogeographic patterns in coastal marine species [17], including macroalgae [18–21]. When the marginal seas in the Northwest Pacific reunited due to postglacial sea-level rise, populations that survived glaciation started to expand outward, with coastal currents (e.g. China Coastal Current and South China Sea Warm Current) possibly helping to accelerate genetic exchange between marine basins [4, 22]. At the same time, the intertidal substrate and/or diluted water may have acted as physical barriers that maintained genetic divergence between isolated populations. For example, the long stretches of salt marshes along Jiangsu Province (Fig. 1), China, likely acted as a geographic barrier to genetic introgression between the YBS and ECS populations in the limpet *Cellana toreuma* [15]. Similar patterns have also been confirmed in rocky intertidal seaweeds along European coasts [23, 24]. As such, empirical surveys concerning population genetic structure of marine species along the coast of China must account for historical elements (e.g. vicariance events) as well as contemporary environmental variables (e.g. oceanic currents, barriers to dispersal).

*Sargassum* is an ecologically dominant alga in much of the Asia-Northwest Pacific (ANP) coastlines, especially in tropics [25]. It is often characterized with high dispersal ability via rafting, as the thalli are easy to break up by waves or grazers to form floating materials, making it a perfected material to discuss how paleoclimatic oscillations and present ocean currents affected the genetic diversity and distribution pattern. For example, *S. muticum* is one of the most well-known invasive species [26, 27], and two clades were detected among the native populations using the *trn*W–I marker: one is widely spread in both native (e.g. Seto Inland Sea) and introduced area (e.g. Canada, UK and France), and the other is restricted along the east coast of Honshu, Japan [2]. The separation of the clades in Pacific-Japan sides may be resulted from the ancient isolation and present restricted gene flow [2]. Similarly, *S. horneri* and *S. hemiphyllum* in the ANP also showed deep genetic split corresponding to the biogeographic basins, and homogeneous populations along the China coasts which ascribe to the founder effects and recent expansion [4]. In contrast, *S. aquifolium* and *S. polycystum* in Southeast Asia demonstrated shallow genetic structure with genetic diversity decline from south to north, implying surviving in a single refugium during glacial period.

*Sargassum thunbergii* Kuntze is a perennial brown macroalga occurring in the intertidal and sublittoral habitats of the ANP, ranging from Sakhalin, Russia, to Hainan Island, China [28]. This species grows abundantly and can form seaweed forests together with other species of *Sargassum* that serve as spawning, nursery, and feeding grounds for marine animals [29]. *S. thunbergii* is well adapted to diverse temperatures and salinities and thus has been proposed as a promising candidate for restoring the structure and function of impacted intertidal zones [30, 31]. In China, however, *S. thunbergii* is one of the most heavily used natural feed for aquaculture animals (e.g. holothurian and abalone), which has led to its near eradication in the past decade. Effective conservation of *S. thunbergii* will depend on detailed biogeographic knowledge of evolutionarily significant units [31–33]. We recently explored population dynamics of *S. thunbergii* across its entire range, and found that the Kuroshio Current played a significant role in shaping population genetic connectivity [22]. Similar diversity patterns were found in the congeneric *S. horneri* [4] but not in *S. fusiforme* in the ANP [16]. Hypervariable genetic markers and genome-scale genotyping have recently shown promise in revealing population genetic differentiation in seaweeds at a micro-geographic scale [18, 34–36], facilitating the exploration of cryptic population structure and biogeographic processes occurring in *S. thunbergii* along the coast of China.

In this study, we integrated microsatellite genotyping and a plastid marker to analyse the diversity and distribution of *S. thunbergii* along the coast of China. Our main goals were to (i) explore fine-scale population genetic connectivity in *S. thunbergii* along the China coast; (ii) determine if the genetic subdivision was influenced by long-distance dispersal at different time scales, and (iii) test which biogeographic scenario summarized above matches the genetic diversity patterns observed in *S. thunbergii* along the China coast.

## Methods

### Sample collection

*Sargassum thunbergii* specimens (*n* = 661) were collected from 22 sites along the coast of China, ranging from 39.04°N to 25.07°N (Additional file 1: Table S1). The Jiangsu Province lacks rock substrate, so no samples were collected in this region. At each site, samples were collected from 25 to 43 individuals at >5 m intervals to maximize spatial representation within a site. Leaf tips (3–5 cm long) were dried and stored in silica gel for molecular analysis.

### Molecular protocol

Genomic DNA was extracted using the Plant genomic DNA kit (Tiangen Biotech CO., Ltd., Beijing, China) according to the manufacturer’s instructions. The plastid RuBisCo spacer (*rbc* spacer) was amplified using the primer set 3F (5′-CATCGTGCTGGTAACTCTAC-3′; [37]) and S97R (5′-CATCTGTCCATTCWACACTAAC-3′; [37]) PCR, electrophoresis, and sequencing were conducted following our previous protocols [22]. The PCR products contain partial *rbc*L gene, partial *rbc*S gene and the spacer region (GenBank accession numbers: MF767271-MF767277), which were included in Additional file 2. Eleven species-specific microsatellites were also used to explore the phylogeographical structure of *S. thunbergii*. Details on the 11 microsatellites are also summarized in Additional file 2. PCR was conducted following the protocol described by [31]. PCR products were analysed on an Applied Biosystems (ABI) 3730xl Genetic Analyzer with Capillary Electrophoresis. Individual genotypes were scored using GENEMAPPER ver. 4.0 (Applied Biosystems) with a size standard (LIZ 500) and an internal control for allele calling; each allele was coded using its size in nucleotides (bp). Microsatellite settings for GENEMAPPER were: default base pair range = 100–250; bin width = 1.3; peak height acceptance, <50 RFU = discard, 50 RFU ≤ h < 200 RFU = check, h ≥ 200 RFU = accept. The presence of null alleles and stutter were assessed using MICROCHECKER 2.23 [38].

### Genetic diversity and variation

For the plastid *rbc* spacer, number of haplotypes (*N*_h_), haplotype (*h*) and nucleotide (*π*) diversity were estimated for each locality using ARLEQUIN 3.5 [39]. This program was also applied to calculate pairwise values of genetic differentiation (*F*_ST_). All results for significance of covariance components were tested using 10^5^ permutations. A median-joining network among plastid haplotypes was constructed using NETWORK 4.5 [40].

For microsatellite loci, allelic richness (*N*_e_), private allelic richness (*A*_p_), inbreeding coefficients (*F*_IS_), mean expected (*H*_e_) and observed (*H*_o_) heterozygosity were calculated in GENETIX 4.05 [41]. Hardy-Weinberg equilibrium (HWE) and linkage disequilibrium (LD) tests were assessed using GENEPOP 4.3 [42]. The significance level for multiple simultaneous comparisons was adjusted using the sequential Bonferroni technique [43]. Pairwise *F*_ST_ and Jost’s *D* (*D*_est_, [44]) were performed using the program SMOGD [45]. The overall *D*_est_ for each population comparison was calculated as the arithmetic mean across loci. The hierarchical analysis of molecular variance (AMOVA) was conducted to detect the proportion of genetic differentiation among regions, and within and among populations [46].

### Microsatellite-based cluster analyses

We used two approaches to infer the number of distinct genetic clusters and to assign individuals to a given cluster. First, a Bayesian clustering analysis was implemented in STRUCTURE 2.3.4 [47, 48]. The program was run with 10 independent simulations for each value of K (= number of clusters) from 1 to 12, each with 800,000 iterations, following a burn-in period of 100,000 iterations. The model assumed admixture, correlated allele frequencies [49] and non-informative priors. The likelihood results were collected and assessed in STRUCTURE HARVESTER [50], using the method ΔK [51]. The results of 10 replicate runs for K were combined in CLUMPP ver.1.1.2 [52], and displayed graphically using DISTRUCT 1.1 [53]. Second, we estimated patterns of population genetic differentiation derived from pairwise *F*_ST_ values with a principal coordinates analysis (PCoA) using GENALEX [41]. To visualize the phylogenetic relationships among samples, an unweighted pair group method with arithmetic mean (UPGMA) dendrogram based on Nei’s genetic distance [54] was constructed using the program TFPGA [55]. Bootstrap analysis (1000 pseudoreplicates) was used to evaluate statistical nodal support.

### Contemporary and historical migration

**Fig. 2:**
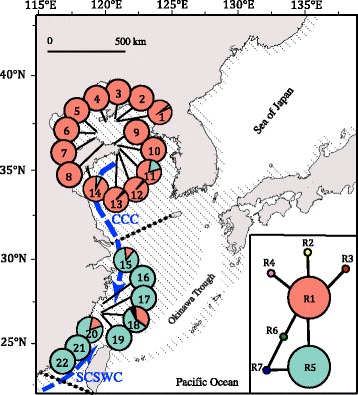
Haplotype distribution along the coast of China and median-joining network inferred from plastid *rbc* spacer data. Each line between main haplotypes represents one mutation step. Detailed locality information is shown in Table 1. Shaded sea areas are continental shelves that would have been exposed to the air during periods of low sea level. CCC: China Coastal Current; SCSWC: South China Sea Warm Current

In order to determine if the population genetic differentiation in *S. thunbergii* along the coast of China was influenced by long-distance dispersal of floating thalli at different time scales, we estimated contemporary and historical gene flow. BAYESASS 3.0 [56] could identify the signature of gene flow from the current generation up to a few past generations. In contrast, MIGRATE-n [57] considers all gene flow between populations from the current generation back to the most recent common ancestor to detect historical processes. Furthermore, in sampling regions, some populations are geographically proximate (e.g. POP1/2/3) (Fig. 2), and in order to facilitate statistical performance we chose one or two populations in each region to conduct following gene flow analyses.

Recent migration rates (*m*) were calculated with BAYESASS 3.0 [56]. It assumes linkage equilibrium and that populations have not been subjected to genetic drift within the past 2–3 generations before sampling, and allows deviations from Hardy-Weinberg expectations within populations [58]. We used the methodology of 3 × 10^6^ iterations, with a burn-in of 10^6^ iterations, and a sampling frequency of 2 × 10^4^. Five replications were performed to ensure consistency between runs.

Mutation-scaled migration rate (M) estimates were calculated using a Bayesian coalescent approach implemented in MIGRATE-n 3.6 [57]. MIGRATE allows deviations from Hardy-Weinberg expectations, but assumes that populations are in migration-drift equilibrium. A Brownian motion model was run to estimate mutation-scaled migration rate *M* (*M* = *m*/*μ*, *m* is historical migration rate, *μ* is mutation rate per generation). Our analyses included 3 long chains and 5 replicates. Burn-in was set in 4 × 10^4^ with a sampling increment of 40 and a total of 5 × 10^4^ recorded steps. Heating was set with four temperatures (1.0, 1.5, 3.0 and 6) with a static scheme. We calculated the effective number of migrants using the relation (*N*_m_ = Θ × *M*/4, Θ is mutation-scaled effective population size) to keep them comparable to contemporary estimates.

## Results

### Plastid DNA

We obtained 661 plastid *rbc* spacer sequences with an aligned length of 795 bp, which contained seven polymorphic sites and yielded seven haplotypes (GenBank accession numbers: MF767271-MF767277). Of these haplotypes, five (R2, R3, R4, R6, R7) were unique and two (R1 and R5) were present in most of the sampling locations (Fig. 2 and Additional file 1: Table S1). Haplotype R1 was mainly detected in the Yellow-Bohai Sea (YBS, 97.9%), whereas haplotype R5 was mainly detected in the East-China Sea (ECS, 90.4%) (Fig. 2 and Additional file 1: Table S1). The populations from Sanpanwei (POP18), Chengshantou (POP11) and Huangqi (POP20) showed the highest genetic diversity (*h* = 0.290–0.551, *π* = 0.037–0.077; Table 1).

**Table 1:** Genetic diversity indices of *Sargassum thunbergii* populations along the coast of China inferred from plastid *rbc* spacer and microsatellites

Sampling localities	*rbc* spacer				microsatellites					
	*N*	*N*_h_	*h*	*π* (×10^−2^)	*N*	*N*_e_	*A*_p_	*H*_o_	*H*_e_	*F*_IS_
1. DongBang, Liaoning, China	26	2	0.077	0.029	25	3.364	0.091	0.302	0.380	0.226
2. Yingzuishi, Liaoning, China	29	1	0.000	0.000	25	3.364	0.182	0.429	0.475	0.117
3. Yazishi, Liaoning, China	35	1	0.000	0.000	25	2.909	0.000	0.404	0.413	0.044
4. Lvshun, Liaoning, China	24	1	0.000	0.000	24	3.000	0.091	0.371	0.399	0.091
5. Beihuangcheng, Yantai, China	34	1	0.000	0.000	25	2.545	0.000	0.302	0.393	0.252
6. Daqin, Yantai, China	34	1	0.000	0.000	25	3.182	0.182	0.345	0.401	0.159
7. Changdao, Yantai, China	30	1	0.000	0.000	25	2.818	0.091	0.302	0.351	0.159
8. Yantai University, China	31	1	0.000	0.000	25	2.545	0.000	0.236	0.243	0.047
9. Xiaoshi Island, Weihai, China	24	1	0.000	0.000	24	2.182	0.000	0.333	0.323	−0.011
10. Jiming Island, Weihai, China	30	1	0.000	0.000	25	3.273	0.091	0.451	0.498	0.114
11. Chengshantou, Weihai, China	22	2	0.312	0.039	25	3.000	0.182	0.411	0.411	0.020
12. Yueliang Bay, Weihai, China	43	2	0.047	0.006	25	2.727	0.091	0.364	0.340	−0.049
13. Ailian Bay, Weihai, China	40	2	0.050	0.006	25	2.909	0.182	0.407	0.437	0.088
14. Badaguan, Qingdao, China	29	2	0.133	0.017	25	2.818	0.182	0.364	0.348	−0.026
15. Shengsi, Zhoushan, China	32	2	0.226	0.028	25	2.545	0.182	0.175	0.212	0.197
16. Dongtou, Wenzhou, China	30	1	0.000	0.000	25	2.909	0.091	0.425	0.419	0.005
17. Longchuanjiao, Wenzhou, China	29	1	0.000	0.000	25	3.000	0.091	0.291	0.309	0.079
18. Sanpanwei, Wenzhou, China	33	4	0.551	0.077	25	2.000	0.091	0.260	0.269	0.054
19. Zhuyu Island, Wenzhou, China	29	1	0.000	0.000	25	2.273	0.455	0.291	0.281	−0.015
20. Huangqi, Fuzhou, China	24	2	0.290	0.037	24	2.455	0.364	0.265	0.253	−0.025
21. Nanri Island, Putian, China	30	1	0.000	0.000	29	2.091	0.182	0.238	0.276	0.154
22. Meizhou Island, Putian, China	23	1	0.000	0.000	24	1.909	0.182	0.227	0.199	−0.122^*^

*N* number of sequences, *N*_h_ number of haplotypes, *h* haplotype diversity, *π* nucleotide diversity, *N*_e_ allelic richness, *A*_p_ private allelic richness, *H*_o_ observed heterozygosity, *H*_e_ expected heterozygosity, *F*_*IS*_ inbreeding coefficient

^*^ *P* < 0.05 (1000 permutations)

### Basic statistics for microsatellites

No evidence of null alleles or stutter was observed in any of the eleven microsatellites studied. Tests for Hardy-Weinberg Equilibrium (HWE) showed little evidence for significant deviations from equilibrium expectations (Additional file 3: Table S3). No evidence of linkage disequilibrium was found. No significant positive values of the inbreeding coefficient *F*_IS_ were detected. Mean allelic richness ranged from 1.909 to 3.364 and the highest value was detected in Dongbang (POP1) and Yingzuishi (POP2). The number of private alleles ranged from 0.000 to 0.182 in the YBS and from 0.091 to 0.455 in the ECS (Table 1), with the highest value detected on Zhuyu Island (POP 19). The populations in the north coast (POP 1–14) (*N*_e_ = 2.182–3.364, *H*_o_ = 0.236–0.451, *H*_e_ = 0.243–0.498) showed slightly higher genetic diversity than those in the south coast (POP 15–22) (*N*_e_ = 1.909–3.000, *H*_o_ = 0.175–0.425, *H*_e_ = 0.212–0.419; Table 1). The highest values of expected and observed heterozygosity were found in Jiming Island (POP10) (*H*_o_ = 0.451, *H*_e_ = 0.498; Table 1).

### Population genetic differentiation and clustering

Pairwise *D*_est_ values revealed high levels of genetic divergence between populations from the YBS and ECS (average *D*_est_ = 0.291; Additional file 4: Figure S1 and Additional file 5: Table S4). In contrast, *D*_est_ estimates were relatively low between populations in the YBS (average *D*_est_ = 0.120) and ECS (average *D*_est_ = 0.047), respectively (Additional file 4: Figure S1 and Additional file 5: Table S4). Similar results were also observed in pairwise *F*_ST_ values based on microsatellites (Additional file 5: Table S4**)**. Pairwise *F*_ST_ values based on *rbc* spacer also indicated deep genetic divergence between the north and south coasts (Additional file 4: Figure S1 and Additional file 6: Table S5). AMOVA analyses based on *rbc* spacer also showed significant divergence between clusters (YBS vs. ECS), accounting for 89.03% of the total variance (Additional file 7: Table S6). However, microsatellite analyses showed that most of the genetic variance occurred within populations (65.38%), despite a deep genetic split detected between two clusters (Φ_CT_ = 0.223, *P* < 0.0001; Additional file 7: Table S6).

**Fig. 3:**
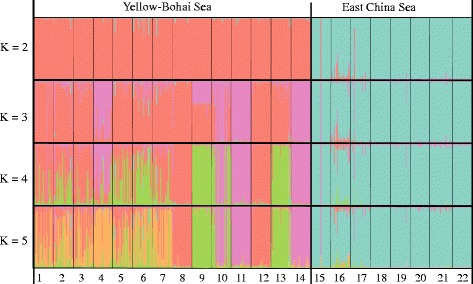
Clustering results of 22 *S. thunbergii* populations based on microsatellites, with K ranging from K = 2 to K = 5. Each individual is depicted by a vertical bar that is partitioned into colored sections. Population codes in parentheses are the same as in Table 1 and Fig. 2

**Fig. 4:**
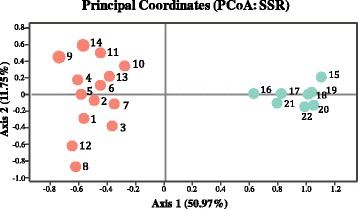
Principal Component Analysis (PCoA) based on microsatellites. The population codes are the same as in Table 1 and Fig. 2

Microsatellite-based clustering results showed that *S. thunbergii* populations were hierarchically structured. The most likely number of genetic clusters was K = 2 (Additional file 8: Figure S2), where *S. thunbergii* individuals in the YBS formed a clade, while individuals in the ECS formed another clade (Fig. 3). We found genetic subdivision of populations in the YBS when K = 3, 4, 5 (Fig. 3). In contrast, no clear population genetic variation was detected on the south coast of China (Fig. 3). A deep genetic split in *S. thunbergii* along the coast of China was also supported by the PCoA profiling in which the north and south coast populations were grouped into the YBS and ECS clusters, respectively (Fig. 4). The UPGMA tree indicated that populations from the YBS and ECS have diverged into two groups with a 100% bootstrap support (Additional file 9: Figure S3).

### Contemporary vs. historical migration

**Fig. 5:**
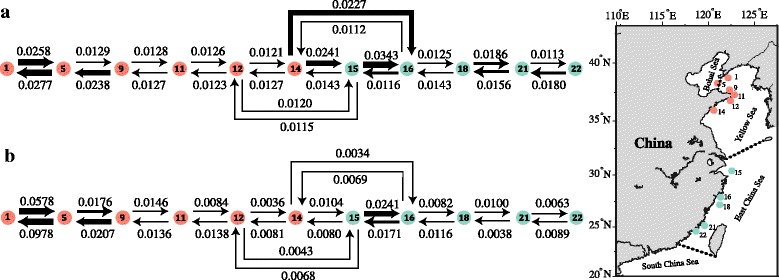
Estimates of contemporary (*m*) (**a**) and historical gene flow (*N*_m_) (**b**) between *S. thunbergii* populations along the coast of China. Numbers above/below arrows represent migration rates in the direction of the arrow. The thickness of arrow is scaled according to the values. Population locations are shown in the map on the right

The five replications in BAYESASS yielded similar results. For populations in the YBS, the highest gene flow was between Dongbang (POP1) and Beihuangcheng (POP5) (*m* = 0.0258, Fig. 5a). Contemporary gene flow between populations in the YBS was mostly symmetrical except for between Beihuangcheng (POP5) and Xiaoshi Island (POP9) (*m* = 0.0129) (Fig. 5a). For ECS populations, the highest contemporary gene flow was from Shengsi (POP15) to Dongtou (POP16) (*m* = 0.0343) and the lowest was from Dongtou (POP16) to Qingdao (POP14) (*m* = 0.0112). In addition, strong asymmetric gene flow was detected from Qingdao (POP14) to Shengsi (POP15) (*m* = 0.0241) and Dongtou (POP16) (*m* = 0.0227), respectively, which was almost two times larger than gene flow in the opposite direction (Fig. 5a). Historical migration rates were the highest from Beihuangcheng (POP5) to Dongbang (POP1) (*N*_m_ = 0.0978) and the lowest from Qingdao (POP14) to Dongtou (POP16) (*N*_m_ = 0.0034; Fig. 5b). When comparing historical to contemporary gene flow, the greatest decline occurred in ECS between Qingdao-Dongtou (POP14 → POP16, *N*_m_ – *m* = −0.0193), followed by Qingdao-Shengsi (POP14 → POP15, *N*_m_ – m = −0.0137; Fig. 5). In contrast, most of the historical gene flow was higher than contemporary estimates in the YBS, and the greatest decline was between Dongbang-Beihuangcheng (POP5 → POP1, *N*_m_ – *m* = 0.0701). All historical gene flow between populations in the ECS were lower than contemporary gene flow except in the pairwise Shengsi-Dongtou (POP16 → POP15; Fig. 5).

## Discussion

In this study, microsatellites and plastid DNA consistently revealed two genetically diverged clusters in *Sargassum thunbergii* along the coast of China, and their geographical distributions are in accordance with two marginal seas: the Yellow-Bohai Sea (YBS) and East China Sea (ECS). In addition, contemporary gene flow driven by coastal currents could cause a low yet significant connectivity between populations in two marine regimes.

### Genetic divergence in *Sargassum thunbergii*

*Sargassum thunbergii* populations were divided into two genetic clusters that correspond to the YBS and ECS (Fig. 2). This biogeographic pattern matches scenario II proposed earlier (see Introduction) along the coast of China, as exemplified in the newly reported congeneric species *S. fusiforme* [16]. Historical vicariance might account for this observed pattern in *S. thunbergii* along the coast of China. Shifting sea levels during the Quaternary ice ages likely caused ancestral populations of *S. thunbergii* to be isolated in multiple refugia [4, 5]. In the ECS, the southern populations (POP19–22) harboured a higher number of private alleles (*A*_p_ = 0.182–0.455) than the northern populations (POP15–18) (*A*_p_ = 0.091–0.182), suggesting that *S. thunbergii* populations in the ECS may have survived in the southern Okinawa Trough or the SCS during glacial periods. Populations in the YBS, on the other hand, were possibly recolonized from the northern Okinawa Trough or the Sea of Japan. In addition, our simulations of gene flow revealed limited historical gene flow between populations from the YBS and ECS (Fig. 5b), strengthening confidence in the putative vicariance event. Comparable postglacial dispersal patterns were also reported in the congeneric species, *S. hemiphyllum* [2] and *S. fusiforme* [16].

Hydrographic factors, such as the Changjiang diluted water (CDW, Fig. 1), have been documented as a major barrier to gene flow in intertidal marine species [15, 59]. Huge freshwater outflow and influx profoundly reduce seawater salinities, pH, and dissolved inorganic nutrients of the ECS, influencing species’ growth and survival [29, 60, 61]. However, recent phylogeographical studies indicated that the hydrological barrier was not insurmountable for some intertidal species with broad temperature and salinity tolerances, such as in *Eriocheir* sensu stricto [1], *Cyclina sinensis* [59], and *S. horneri* [4]. *Sargassum thunbergii* has a broad tolerance to salinity (12–34 psu; [29]) which exceeds the sea surface water salinity range of the Changjiang River Estuary during summer (15–31 psu; [62]), thus enabling it to cross the physical barrier. More importantly, research cruises reported that *Sargassum* beds (including *S. thunbergii*) could be transported to fringe areas of the continental shelf and waters influenced by the Kuroshio Current after they become detached off Zhejiang Province, China [63, 64]. We thus infer that the CDW did not impede gene flow between *S. thunbergii* populations in the YBS and ECS respectively, an assumption that was further supported by shared haplotypes (Fig. 2) and contemporary gene flow between the two regions (Fig. 5a). Similarly, a marine biogeographic break at 30°S was detected in most intertidal species along the coast of the temperate South East Pacific (SEP) [65, 66], and the upwelling seems to be the dominant factor influencing coastal communities. While some coastal species, such as *Stichaster striatus* and *Tegula atra*, showed low yet significant northward migration across the 30°S break, which have been able to achieve significant connectivity between biogeographic regions, slightly eroding the historic signature [66]. Thus dispersal potential and the levels of tolerance was the variable that always best explained the contrasting genetic structure of co-distributed species.

### Ocean currents, coastal barriers and population connectivity

Patterns in genetic diversity inferred from Quaternary glaciation events may be obscured by environmental factors. In this study, the two genetic lineages of *S. thunbergii* along the coast of China may indicate different evolutionary processes. We detected substantial population subdivision in the YBS, but no clear genetic divergence among populations in the ECS (Figs 4 and 5), a scenario resembling the phylogeographical structure observed in *S. fusiforme* [16]. The two contrasting patterns may be ascribed to different environmental variables and coastal topographies. The coastline of the YBS includes the Bohai Bay and Shandong peninsula, which may act as geographic barriers for genetic exchange between populations [67, 68], leading to the formation of substructure. In contrast, the coastline of the ECS lacks geographical or physical barriers. Thus, the population structuring of *S. thunbergii* along the ECS was mainly dictated by coastal currents, particularly during the monsoon seasons [69]. Under the southwest monsoon, the water in the Taiwan Strait originates mainly from the South China Sea Warm Current (summer) and the Taiwan Warm Current (spring) [70]. These monsoon-driven coastal currents can facilitate long-distance dispersal of floating *S. thunbergii*, leading to population homogenization in the ECS (Fig. 5a; [18]). Oceanographic circulations have also caused the contrasting phylogeographic patterns between coastal species in the Japan-Pacific and the Sea of Japan. Greater dispersal potential exists in the Sea of Japan because of the Tsushima Warm Current, whereas there is comparatively limited dispersal potential by drifting along the Japan-Pacific coasts owing to the complex oceanographic circulations (e.g. southward-flowing Tsugaru Current and northward-flowing Kuroshio Current) and multiple presenting hydrographic barriers to dispersal [16, 22].

Interestingly, our results indicated that *S. thunbergii* populations in the YBS (northern area) had higher genetic diversity than in the ECS (southern area) (Table 1). Similar results were detected in the limpet *Cellana toreuma* in this region [15]. This pattern is clearly different from that reported in most intertidal species along European coastlines which show a characteristic reduction in genetic diversity with increasing latitude as major refugial areas occurred at low latitudes [24]. High genetic diversity and significantly large pairwise genetic distance support the conclusion that the YBS group was largely isolated from the ECS and the potential refugia located at marginal area other than southern area. Alternatively, the Yellow Sea Warm Current may have bring individuals of *S. thunbergii* into the YBS from the west coast of the Korean Peninsula (e.g. the Jeju Island), leading to high levels of genetic admixture as reported in the red alga *Gelidium elegans* [71].

## Conclusions

The genetic divergence of *S. thunbergii* in the YBS and the ECS suggest long-term isolation. Besides, the comparison of contemporary and historical gene flow indicate that the Changjiang diluted water could not restrict the gene flow of *S. thunbegii* populations along the Chinese coasts. These findings contrast with some previous studies on invertebrates with similar phylogeographic structure [10, 15]. Future phylogeoghic studies in this region should not only focus on establishing patterns of genetic divergence in intertidal species, but also consider fine-scale patterns of gene flow using multilocus markers. The interpretation of patterns of phylogeographic structure and genetic exchange could provide a framework for marine biodiversity conservation.

## Additional files

Additional file 1: Table S1. Sampling locality, code, coordinates, sample size and RuBisCo spacer (*rbc* spacer) haplotype distribution in each *Sargassum thunbergii* population. (DOCX 15 kb)

Additional file 2: Table S2. Sequences of 7 *rbc* spacer haplotypes and primers of 11 microsatellite markers. (DOCX 15 kb)

Additional file 3: Table S3. Probability of deviation from Hardy-Weinberg equilibrium (*p*) for each population and each locus. (DOCX 19 kb)

Additional file 4: Figure S1. The average *D*_est_ matrix based on microsatellites (upper right) and *F*_ST_ based on plastid *rbc* spacer (lower left), respectively. (DOCX 113 kb)

Additional file 5: Table S4. Pairwise differentiation of 22 *Sargassum thunbergii* populations base on microsatellites. (DOCX 18 kb)

Additional file 6: Table S5. *F*_ST_ values between 22 *Sargassum thunbergii* populations based plastid *rbc* spacer. (DOCX 16 kb)

Additional file 7: Table S6. Analysis of molecular variance (AMOVA) to partition genetic variance in the Northwest Pacific *Sargassum thunbergii* based on *rbc* spacer and SSR. (DOCX 14 kb)

Additional file 8: Figure S2. Most likely number of genetic clusters using the Evanno method for *Sargassum thunbergii* individuals using 11 microsatellites from all the localities along the coast of China. (DOCX 51 kb)

Additional file 9: Figure S3. UPGMA tree of 22 *Sargassum thunbergii* populations based on microsatellites. (DOCX 53 kb)

## Abbreviations

ANP: Asia-Northwest Pacific
CDW: Changjiang diluted water
ECS: East China Sea
HWE: Hardy-Weinberg Equilibrium
LGM: Last Glacial Maximum
SCS: South China Sea
SEP: South East Pacific
SOJ: Sea of Japan
YBS: Yellow-Bohai Sea
