# Supplementary material for: Historical isolation and contemporary gene flow drive population diversity of the brown alga Sargassum thunbergii along the coast of China
Source: BMC Evol Biol. 2017 Dec 7;17:246. doi: 10.1186/s12862-017-1089-6 (PMC5721624; doi:10.1186/s12862-017-1089-6)
Supplement: Supplementary file 1 — Sampling locality, code, coordinates, sample size and RuBisCo spacer (rbc spacer) haplotype distribution in each Sargassum thunbergii population. (DOCX 15 kb) [file 12862_2017_1089_MOESM1_ESM.docx]

**Additional file 1: Table S1:** Sampling locality, code, coordinates, sample size and RuBisCo spacer (*rbc* spacer) haplotype distribution in each *Sargassum thunbergii* population. The corresponding codes are the same as in Table 1 and Fig. 2.

| Sampling locality | Coordinate | Sample size | *rbc*L-S |
| --- | --- | --- | --- |
| 1. DongBang, Liaoning, China | 39.02°N, 122.75°E | 26 | R1(25), R2(1) |
| 2. Yingzuishi, Liaoning, China | 39.01°N, 122.73°E | 29 | R1(29) |
| 3. Yazishi, Liaoning, China | 39.04°N, 122.72°E | 35 | R1(35) |
| 4. Lvshun, Liaoning, China | 38.72°N, 121.15°E | 24 | R1(24) |
| 5. Beihuangcheng, Yantai, China | 38.38°N, 120.90°E | 34 | R1(34) |
| 6. Daqin, Yantai, China | 38.30°N, 120.83°E | 34 | R1(34) |
| 7. Changdao, Yantai, China | 37.97°N, 120.73°E | 30 | R1(30) |
| 8. Yantai University, China | 37.47°N, 121.46°E | 31 | R1(31) |
| 9. Xiaoshi Island, Weihai, China | 37.52°N, 122.01°E | 24 | R1(24) |
| 10. Jiming Island, Weihai, China | 37.75°N, 122.80°E | 30 | R1(30) |
| 11. Chengshantou, Weihai, China | 37.39°N, 122.71°E | 22 | R1(18), R5(4) |
| 12. Yueliang Bay, Weihai, China | 37.51°N, 122.43°E | 43 | R1(42), R3(1) |
| 13. Ailian Bay, Weihai, China | 37.23°N, 122.59°E | 40 | R1(39), R4(1) |
| 14. Qingdao, China | 36.05°N, 120.35°E | 29 | R1(27), R5(2) |
| 15. Shengsi, Zhoushan, China | 30.71°N, 122.45°E | 32 | R1(4), R5(28) |
| 16. Dongtou, Wenzhou, China | 27.80°N, 121.14°E | 30 | R5(30) |
| 17. Longchuanjiao, Wenzhou, China | 27.45°N, 121.05°E | 29 | R5(29) |
| 18. Sanpanwei, Wenzhou, China | 27.48°N, 121.05°E | 33 | R1(12), R5(19), R6(1), R7(1) |
| 19. Zhuyu Island, Wenzhou, China | 27.46°N, 121.10°E | 29 | R5(29) |
| 20. Huangqi, Fuzhou, China | 26.41°N, 119.92°E | 24 | R1(4), R5(20) |
| 21. Nanri Island, Putian, China | 25.26°N, 119.67°E | 30 | R5(30) |
| 22. Meizhou Island, Putian, China | 25.07°N, 119.13°E | 23 | R5(23) |
