# Supplementary material for: Historical isolation and contemporary gene flow drive population diversity of the brown alga Sargassum thunbergii along the coast of China
Source: BMC Evol Biol. 2017 Dec 7;17:246. doi: 10.1186/s12862-017-1089-6 (PMC5721624; doi:10.1186/s12862-017-1089-6)
Supplement: Supplementary file 2 — Sequences of 7 rbc spacer haplotypes and primers of 11 microsatellite markers. (DOCX 15 kb) [file 12862_2017_1089_MOESM2_ESM.docx]

**Additional file 2: Table S2** Sequences of 7 *rbc* spacer haplotypes and primers of 11 microsatellite markers. Ta: annealing temperature

| Haplotype | Sequences |
| --- | --- |
| R1 | TATTAACTTTAGGGTTATTTGTAAATGGATGCGTATGTCTGGTGTAGATCATATTCATGCAGGTACAGTTGTAGGGAAACTAGAAGGAGATCCTTTGATGGTTAGAGGATTTTATAATACACTATTGTTAACTGAATTAAAAATTAATTTAGCTGAAGGACTATTTTTCGATATGGATTGGGCATCGCTCCGAAAATGTGTTCCTGTAGCATCAGGTGGTATTCATTGTGGTCAAATGCATCAACTTCTTTATTATTTAGGCGATGATGTTGTTCTACAATTTGGAGGTGGTACAATTGGTCACCCTGATGGTATACAAGCAGGTGCGACAGCAAATCGTGTTGCTTTAGAAGCAATGGTTCTAGCTAGAAATGAGGGTCGTGATTATGTTGGTGAAGGACCGGAAATTTTACGTACTGCGGCAAGTACTTGTGGACCGTTAAAAGCAGCTTTAGATCTATGGAAAGATATTACTTTTGAATATACTTCAACAGACACACCTGATTTTGTTGAAGTAGCGACTGAAAGTCCTTAAATTTATTCTATTCTATAGTGTAATTTTTACTATAAAAGAACAGACTAATGAATTTTAATCTTTTTAATATTTTATATTAAGACAAAAGATAGAAAAAGTTTAGTAGTTAACTAAAAATAAAATTAAAATATTTACATTAAATAAAATAATTGAAGAGTAATGAGACTTACACAAGGATGTTTTTCATTTTTACCAGATTTAAGTGACGAACAAATTAAACATCAAGTTGGTTATGCTATTTCAAAAGGATGGGCCGTTAG |
| R2 | TATTAACTTTAGGGTTATTTGTAAATGGATGCGTATGTCTGGTGTAGATCATATTCATGCAGGTACAGTTGTAGGGAAACTAGAAGGAGATCCTTTGATGGTTAGAGGATTTTATAATACCCTATTGTTAACTGAATTAAAAATTAATTTAGCTGAAGGACTATTTTTCGATATGGATTGGGCATCGCTCCGAAAATGTGTTCCTGTAGCATCAGGTGGTATTCATTGTGGTCAAATGCATCAACTTCTTTATTATTTAGGCGATGATGTTGTTCTACAATTTGGAGGTGGTACAATTGGTCACCCTGATGGTATACAAGCAGGTGCGACAGCAAATCGTGTTGCTTTAGAAGCAATGGTTCTAGCTAGAAATGAGGGTCGTGATTATGTTGGTGAAGGACCGGAAATTTTACGTACTGCGGCAAGTACTTGTGGACCGTTAAAAGCAGCTTTAGATCTATGGAAAGATATTACTTTTGAATATACTTCAACAGACACACCTGATTTTGTTGAAGTAGCGACTGACAGTCCTTAAATTTATTCTATTCTATAGTGTAATTTTTACTATAAAAGAACAGACTAATGAATTTTAATCTTTTTAATATTTTATATTAAGACAAAAGATAGAAAAAGTTTAGTACTTAACTAAAAATAAAATTAAAATATTTACATTAAATAAAATAATTGAAGAGTAATGAGACTTACACAAGGATGTTTTTCATTTTTACCAGATTTAAGTGACGAACAAATTAAACATCAAGTTGGTTATGCTATTTCAAAAGGATGGGCCGTTAG |
| R3 | TATTAACTTTAGGGTTATTTGTAAATGGATGCGTATGTCTGGTGTAGATCATATTCATGCAGGTACAGTTGTAGGGAAACTAGAAGGAGATCCTTTGATGGTTAGAGGATTTTATAATACACTATTGTTAACTGAATTAAAAATTAATTTAGCTGAAGGACTATTTTTCGATATGGATTGGGCATCGCTCCGAAAATGTGTTCCTGTAGCATCAGGTGGTATTCATTGTGGTCAAATGCATCAACTTCTTTATTATTTAGGCGATGATGTTGTTCTACAATTTGGAGGTGGTACAATTGGTCACCCTGATGGTATACAAGCAGGTGCGACAGCAAATCGTGTTGCTTTAGAAGCAATGGTTCTAGCTAGAAATGAGGGTCGTGATTATGTTGGTGAAGGACCGGAAATTTTACGTACTGCGGCAAGTACTTGTGGACCGTTAAAAGCAGCTTTAGATCTATGGAAAGATATTACTTTTGAATATACTTCAACAGACACACCTGATTTTGTTGAAGTAGCGACTGAAAGTCCTTAAATTTATTCTATTCTATAGTGTAATTTTTACTATAAAAGAACAGACTAATGAATTTTAATCTTTTTAATATTTTATATTAAGACAAAAGATAGAAAAAGTTTAGTAGTTAACTAAAAATAAAATTAAAATATTTACATTAAATAAAATAATTGAAGAGTAATGAGACTTACACAAGGATGTTTTTCATTTTTACCTGATTTAAGTGACGAACAAATTAAACATCAAGTTGGTTATGCTATTTCAAAAGGATGGGCCGTTAG |
| R4 | TATTAACTTTAGGGTTATTTGTAAATGGATGCGTATGTCTGGTGTAGATCATATTCATGCAGGTACAGTTGTAGGGAAACTAGAAGGAGATCCTTTGATGGTTAGAGGATTTTATAATACACTATTGTTAACTGAATTAAAAATTAATTTAGCTGAAGGACTATTTTTCGATATGGATTGGGCATCGCTCCGAAAATGTGTTCCTGTAGCATCAGGTGGTATTCATTGTGGTCAAATGCATCAACTTCTTTATTATTTAGGCGATGATGTTGTTCTACAATTTGGAGGTGGTACAATTGGTCACCCTGATGGTATACAAGCAGGTGCGACAGCAAATCGTGTTGCTTTAGAAGCAATGGTTCTAGCTAGAAATGAGGGTCGTGATTATGTTGGTGAAGGACCGGAAATTTTACGTACTGCGGCAAGTACTTGTGGACCGTTAAAAGCAGCTTTAGATCTATGGAAAGATATTACTTTTGAATATACTTCAACAGACACACCTGATTTTGTTGAAGTAGCGACTGAAAGTCCTTAAATTTATTCTATTCTATAGTGTAATTTTTACTATAAAAGAACAGACTAATGAATTTTAATCTTTTTAATATTTTATATTAAGACAAAAGATAGAAAAAGTTTAGTAGTTAACTAAAAATAAAATTAAAATATTTACATTAAATAAAATAATTGAAGAGTAATGAGACTTACACAAGGATGTTTTTCATTTTTACCAGATTTAAGTGACGAACAAATTAAACATCAAGTTGGATATGCTATTTCAAAAGGATGGGCCGTTAG |
| R5 | TATTAACTTTAGGGTTATTTGTAAATGGATGCGTATGTCTGGTGTAGATCATATTCATGCAGGTACAGTTGTAGGGAAACTAGAAGGAGATCCTTTGATGGTTAGAGGATTTTATAATACACTATTGTTAACTGAATTAAAAATTAATTTAGCTGAAGGACTATTTTTCGATATGGATTGGGCATCGCTCCGAAAATGTGTTCCTGTAGCATCAGGTGGTATTCATTGTGGTCAAATGCATCAACTTCTTTATTATTTAGGCGATGATGTTGTTCTACAATTTGGAGGTGGTACAATTGGTCACCCTGATGGTATACAAGCAGGTGCGACAGCAAATCGTGTTGCTTTAGAAGCTATGGTTCTAGCTAGAAATGAGGGTCGTGATTATGTTGGTGAAGGACCGGAAATTTTACGTACTGCGGCAAGTACTTGTGGACCGTTAAAAGCAGCTTTAGATCTATGGAAAGATATTACTTTTGAATATACTTCAACAGACACACCTGATTTTGTTGAAGTAGCGACTGAAAGTCCTTAAATTTATTCTATTCTATAGTGTAATTTTTACTATAAAAGAACAGACTAATGAATTTTAATCTTTTTAATATTTTATATTAAGACAAAAGATAGAAAAAGTTTAGTAGTTAACTAAAAATAAAATTAAAATATTTACATTAAATAAAATAATTGAAGAGTAATGAGACTTACACAAGGATGTTTTTCATTTTTACCAGATTTAAGTGACGAACAAATTAAACATCAAGTTGGTTATGCTATTTCAAAAGGATGGGCCGTTAG |
| R6 | TATTAACTTTAGGGTTATTTGTAAATGGATGCGTATGTCTGGTGTAGATCATATTCATGCAGGTACAGTTGTAGGGAAACTAGAAGGAGATCCTTTGATGGTTAGAGGATTTTATAATACACTATTGTTAACTGAATTAAAAATTAATTTAGCTGAAGGACTATTTTTCGATATGGATTGGGCATCGCTCCGAAAATGTGTTCCTGTAGCATCAGGTGGTATTCATTGTGGTCAAATGCATCAACTTCTTTATTATTTAGGCGATGATGTTGTTCTACAATTTGGAGGTGGTACAATTGGTCACCCTGATGGTATACAAGCAGGTGCGACAGCAAATCGTGTTGCTTTAGAAGCAATGGTTCTAGCTAGAAATGAGGGTCGTGATTATGTTGGTGAAGGACCGGAAATTTTACGTACTGCGGCAAGTACTTGTGGACCGTTAAAAGCAGCTTTAGATCTATGGAAAGATATTACTTTTGAATATACTTCAACAGACACACCTGATTTTGTTGAAGTAGCGACTGAAAGTCCTTAAATTTATTCTATTCTATAGTGTAATTTTTACTATAAAAGAACAGACTAATGAATTTTAATCTTTTTAATATTTTATATTAAGACAAAAGATAGAAAAAGTTTAGTAGTTAACTAAAAATAAAATTAAAATATTTACATTAAATAAAATAATTGAAGAGTAATGAGACTTACACAAGGATGTTTTTCATTTTTATCAGATTTAAGTGACGAACAAATTAAACATCAAGTTGGTTATGCTATTTCAAAAGGATGGGCCGTTAG |
| R7 | TATTAACTTTAGGGTTATTTGTAAATGGATGCGTATGTCTGGTGTAGATCATATTCATGCAGGTACAGTTGTAGGGAAACTAGAAGGAGATCCTTTGATGGTTAGAGGATTTTATAATACACTATTGTTAACTGAATTAAAAATTAATTTAGCTGAAGGACTATTTTTCGATATGGATTGGGCATCGCTCCGAAAATGTGTTCCTGTAGCATCAGGTGGTATTCATTGTGGTCAAATGCATCAACTTCTTTATTATTTAGGCGATGATGTTGTTCTACAATTTGGAGGTGGTACAATTGGTCACCCTGATGGTATACAAGCAGGTGCGACAGCAAATCGTGTTGCTTTAGAAGCTATGGTTCTAGCTAGAAATGAGGGTCGTGATTATGTTGGTGAAGGACCGGAAATTTTACGTACTGCGGCAAGTACTTGTGGACCGTTAAAAGCAGCTTTAGATCTATGGAAAGATATTACTTTTGAATATACTTCAACAGACACACCTGATTTTGTTGAAGTAGCGACTGAAAGTCCTTAAATTTATTCTATTCTATAGTGTAATTTTTACTATAAAAGAACAGACTAATGAATTTTAATCTTTTTAATATTTTATATTAAGACAAAAGATAGAAAAAGTTTAGTAGTTAACTAAAAATAAAATTAAAATATTTACATTAAATAAAATAATTGAAGAGTAATGAGACTTACACAAGGATGTTTTTCATTTTTATCAGATTTAAGTGACGAACAAATTAAACATCAAGTTGGTTATGCTATTTCAAAAGGATGGGCCGTTAG |

| Loci | Primer sequences (5’-3’) | Repeat motif | Ta (℃) |
| --- | --- | --- | --- |
| SW1 | AACGGAAGCGCAATACGAC | (AC)_11_ | 60 |
|  | GCAGACACGGTTGACGAAG |  |  |
| SW6 | CAAAGTTGCTGCGTGATTCG | (CT)_11_ | 60 |
|  | CACGATGTGTCGCCTTCTG |  |  |
| SW9 | AAAGTTGCTGAGCCGTTCG | (ACC)_8_ | 60 |
|  | CAGGAGGACCATCGATCCC |  |  |
| SW10 | TGGCTGTGTGGATACGACC | (ACT)_8_ | 60 |
|  | TGTCGCAATGCTCGTTGTAG |  |  |
| SW16 | CCCAAATCAGCGAAAGGCG | (GTT)_21_ | 61 |
|  | CGGTGCTACGATACTGCCC |  |  |
| SW17 | GCCTTCGTTACGCTTGACC | (ATTT)_6_ | 60 |
|  | TACCACCTGAGCAATCCCG |  |  |
| SW18 | ACCCGACGAGCTCTACAAG | (CAGT)_6_ | 60 |
|  | TGAGTGGGTTGAAGACGGG |  |  |
| SW21 | ATGCCAGGAGCTACACAGG | (AAACC)_7_ | 60 |
|  | AGATGGCTCAACCTCTGCC |  |  |
| SW24 | TTGCCCGGGTATCCTGTTC | (AAACAT)_4_ | 60 |
|  | TTTCGCGTTGAGCACTTCG |  |  |
| SW28 | GAGTTTGAGGCGACGGAAG | (AGGATC)_4_ | 59 |
|  | TGCCCTGACTACGCTACAG |  |  |
| SW35 | GCTATGTCAACAACCACCTCT | (ATC)_4_…(ATC)_4_ | 59 |
|  | TTCTGATTCGAGGTATTGTGC |  |  |
