# Supplementary material for: Historical isolation and contemporary gene flow drive population diversity of the brown alga Sargassum thunbergii along the coast of China
Source: BMC Evol Biol. 2017 Dec 7;17:246. doi: 10.1186/s12862-017-1089-6 (PMC5721624; doi:10.1186/s12862-017-1089-6)
Supplement: Supplementary file 3 — Probability of deviation from Hardy-Weinberg equilibrium (p) for each population and each locus. (DOCX 19 kb) [file 12862_2017_1089_MOESM3_ESM.docx]

**Additional file 3: Table S3:** Probability of deviation from Hardy-Weinberg equilibrium (*p*) for each population and each locus. MO: Monomorphic; *p* in bold indicate significant. Population codes are the same as Table S1.

|  | 1 | 2 | 3 | 4 | 5 | 6 | 7 | 8 | 9 | 10 | 11 | 12 | 13 | 14 | 15 | 16 | 17 | 18 | 19 | 20 | 21 | 22 |
| --- | --- | --- | --- | --- | --- | --- | --- | --- | --- | --- | --- | --- | --- | --- | --- | --- | --- | --- | --- | --- | --- | --- |
| SW1 | **0.000** | 0.909 | 0.590 | 0.223 | **0.005** | **0.000** | **0.008** | 0.611 | 0.258 | 0.944 | 0.416 | 0.572 | 0.172 | 0.872 | **0.000** | 0.668 | **0.000** | 0.835 | **0.000** | 0.686 | 0.611 | MO |
| SW6 | **0.000** | 0.779 | 1.000 | 0.258 | 0.629 | **0.000** | 0.579 | 0.664 | 0.367 | 0.841 | 0.152 | 0.579 | 0.848 | 0.919 | 0.919 | 0.664 | **0.003** | 0.835 | 1.000 | 0.422 | 0.925 | MO |
| SW16 | 0.835 | 0.664 | **0.010** | 0.921 | **0.012** | 0.922 | 0.407 | 0.579 | 0.689 | 0.341 | 0.927 | 0.882 | 0.114 | 0.579 | 0.992 | 0.158 | 0.559 | **0.048** | 0.052 | **0.003** | **0.000** | 0.917 |
| SW17 | **0.000** | **0.022** | **0.000** | 0.921 | 0.169 | 0.732 | 0.835 | 0.592 | 0.446 | **0.010** | 0.052 | **0.002** | **0.000** | 0.348 | **0.000** | MO | **0.000** | MO | MO | 0.917 | 0.999 | 0.917 |
| SW18 | **0.000** | **0.000** | 0.299 | 0.197 | 0.919 | 0.117 | 0.416 | MO | MO | 0.979 | **0.000** | MO | **0.000** | 0.628 | 0.919 | 0.390 | **0.000** | 0.060 | **0.043** | 0.102 | MO | MO |
| SW24 | **0.000** | **0.003** | **0.001** | **0.001** | 0.405 | 0.114 | 0.317 | 1.000 | 0.586 | 0.320 | 0.065 | 0.092 | 0.628 | **0.043** | MO | 0.919 | MO | MO | MO | 0.127 | **0.020** | MO |
| SW28 | 0.951 | 0.058 | **0.000** | 0.221 | **0.000** | **0.000** | **0.000** | **0.000** | 0.917 | **0.000** | **0.000** | 0.198 | **0.000** | **0.001** | **0.000** | **0.000** | **0.025** | 0.323 | **0.000** | 0.997 | MO | **0.000** |
| SW35 | 0.992 | 0.341 | 0.835 | 0.917 | 0.495 | 0.750 | MO | 0.835 | **0.010** | 0.211 | **0.004** | 0.495 | 0.397 | 0.741 | **0.000** | **0.000** | 0.643 | 0.804 | 0.405 | 0.656 | 0.785 | MO |
| SW9 | 0.326 | 0.202 | 0.324 | 0.094 | 0.429 | 0.998 | 0.108 | 0.884 | 0.744 | 0.465 | 0.638 | 0.724 | 0.245 | 0.630 | **0.000** | 0.341 | 0.919 | 0.919 | MO | MO | 0.573 | MO |
| SW10 | 0.666 | 0.860 | **0.000** | **0.001** | 0.596 | 0.859 | 0.111 | 0.992 | 0.963 | 0.367 | **0.000** | 0.882 | 0.057 | **0.000** | 0.061 | 0.224 | **0.000** | 0.903 | 0.821 | 0.997 | 0.160 | 0.112 |
| SW21 | 0.740 | 0.338 | **0.019** | 0.160 | 0.358 | 0.394 | 0.970 | 0.882 | 0.392 | 0.464 | 0.473 | 0.608 | 0.835 | 0.416 | 0.979 | 0.918 | 0.272 | 0.841 | **0.003** | 0.698 | **0.020** | **0.002** |
