# Supplementary material for: Historical isolation and contemporary gene flow drive population diversity of the brown alga Sargassum thunbergii along the coast of China
Source: BMC Evol Biol. 2017 Dec 7;17:246. doi: 10.1186/s12862-017-1089-6 (PMC5721624; doi:10.1186/s12862-017-1089-6)

**Additional file 4: Fig. S1:** The average *D*_est_ matrix based on microsatellites (upper right) and *F*_ST_ based on plastid *rbc* spacer (lower left), respectively. Red color indicates a larger value while light blue indicates a small one. Population codes in parentheses are the same as in Table 1 and Fig. 2.


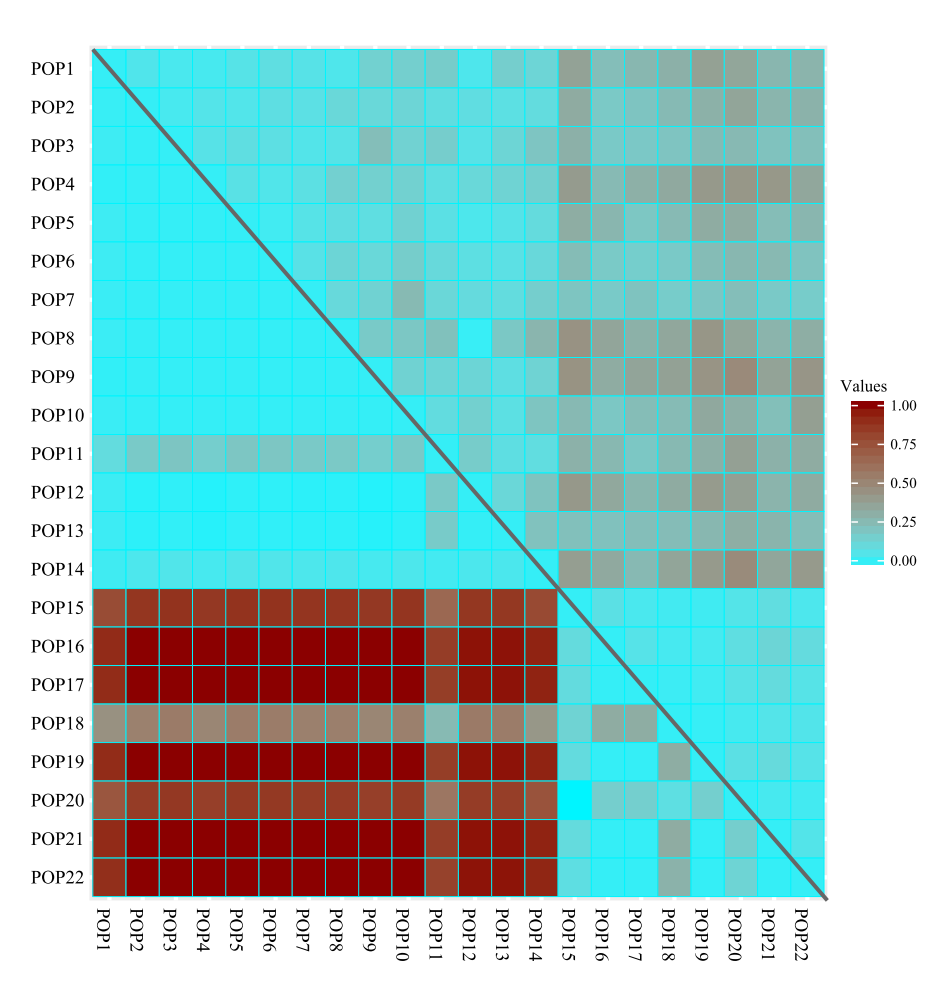

Supplement: Supplementary file 4 — The average D est matrix based on microsatellites (upper right) and F ST based on plastid rbc spacer (lower left), respectively. (DOCX 113 kb) [file 12862_2017_1089_MOESM4_ESM.docx]
