# Supplementary material for: Historical isolation and contemporary gene flow drive population diversity of the brown alga Sargassum thunbergii along the coast of China
Source: BMC Evol Biol. 2017 Dec 7;17:246. doi: 10.1186/s12862-017-1089-6 (PMC5721624; doi:10.1186/s12862-017-1089-6)
Supplement: Supplementary file 5 — Pairwise differentiation of 22 Sargassum thunbergii populations base on microsatellites. (DOCX 18 kb) [file 12862_2017_1089_MOESM5_ESM.docx]

**Additional file 5: Table S4:** Pairwise differentiation of 22 *Sargassum thunbergii* populations base on microsatellites. Jost’s *D*_est_ are given below diagonal. *F*_ST_ values were given above diagonal and all the values are significant (*p* < 0.01). Values inside box indicated *D*_est_ and *F*_ST_ between populations in the Yellow-Bohai Sea and East China Sea.

|  | 1 | 2 | 3 | 4 | 5 | 6 | 7 | 8 | 9 | 10 | 11 | 12 | 13 | 14 | 15 | 16 | 17 | 18 | 19 | 20 | 21 | 22 |
| --- | --- | --- | --- | --- | --- | --- | --- | --- | --- | --- | --- | --- | --- | --- | --- | --- | --- | --- | --- | --- | --- | --- |
| 1 |  | 0.092 | 0.101 | 0.187 | 0.140 | 0.154 | 0.187 | 0.245 | 0.315 | 0.244 | 0.302 | 0.203 | 0.261 | 0.308 | 0.541 | 0.319 | 0.427 | 0.467 | 0.482 | 0.521 | 0.463 | 0.522 |
| 2 | 0.043 |  | 0.109 | 0.196 | 0.102 | 0.144 | 0.16 | 0.252 | 0.267 | 0.177 | 0.194 | 0.172 | 0.181 | 0.225 | 0.474 | 0.279 | 0.350 | 0.400 | 0.415 | 0.451 | 0.412 | 0.480 |
| 3 | 0.030 | 0.027 |  | 0.222 | 0.179 | 0.183 | 0.187 | 0.250 | 0.389 | 0.237 | 0.291 | 0.214 | 0.215 | 0.336 | 0.498 | 0.283 | 0.383 | 0.423 | 0.429 | 0.464 | 0.431 | 0.458 |
| 4 | 0.026 | 0.050 | 0.057 |  | 0.212 | 0.175 | 0.270 | 0.384 | 0.323 | 0.226 | 0.205 | 0.311 | 0.270 | 0.317 | 0.557 | 0.366 | 0.452 | 0.498 | 0.511 | 0.550 | 0.519 | 0.539 |
| 5 | 0.056 | 0.046 | 0.078 | 0.063 |  | 0.059 | 0.158 | 0.294 | 0.263 | 0.227 | 0.205 | 0.187 | 0.162 | 0.238 | 0.516 | 0.359 | 0.393 | 0.455 | 0.475 | 0.498 | 0.449 | 0.512 |
| 6 | 0.078 | 0.075 | 0.072 | 0.044 | 0.017 |  | 0.134 | 0.348 | 0.261 | 0.250 | 0.226 | 0.265 | 0.172 | 0.248 | 0.482 | 0.317 | 0.374 | 0.422 | 0.436 | 0.483 | 0.456 | 0.464 |
| 7 | 0.058 | 0.069 | 0.049 | 0.070 | 0.055 | 0.061 |  | 0.356 | 0.363 | 0.325 | 0.278 | 0.288 | 0.229 | 0.319 | 0.503 | 0.345 | 0.435 | 0.451 | 0.456 | 0.505 | 0.458 | 0.467 |
| 8 | 0.041 | 0.108 | 0.060 | 0.143 | 0.080 | 0.118 | 0.101 |  | 0.476 | 0.364 | 0.418 | 0.029 | 0.383 | 0.507 | 0.666 | 0.474 | 0.550 | 0.596 | 0.611 | 0.612 | 0.572 | 0.638 |
| 9 | 0.141 | 0.108 | 0.229 | 0.144 | 0.077 | 0.107 | 0.133 | 0.170 |  | 0.328 | 0.373 | 0.213 | 0.375 | 0.588 | 0.458 | 0.534 | 0.571 | 0.579 | 0.610 | 0.570 | 0.628 | 0.328 |
| 10 | 0.148 | 0.121 | 0.135 | 0.141 | 0.138 | 0.159 | 0.251 | 0.185 | 0.133 |  | 0.160 | 0.275 | 0.171 | 0.286 | 0.406 | 0.252 | 0.356 | 0.391 | 0.419 | 0.421 | 0.379 | 0.471 |
| 11 | 0.163 | 0.081 | 0.158 | 0.078 | 0.066 | 0.102 | 0.112 | 0.216 | 0.120 | 0.087 |  | 0.322 | 0.242 | 0.225 | 0.515 | 0.370 | 0.415 | 0.467 | 0.484 | 0.510 | 0.473 | 0.532 |
| 12 | 0.039 | 0.080 | 0.061 | 0.108 | 0.037 | 0.073 | 0.095 | 0.003 | 0.118 | 0.145 | 0.163 |  | 0.284 | 0.404 | 0.601 | 0.421 | 0.480 | 0.536 | 0.555 | 0.550 | 0.508 | 0.581 |
| 13 | 0.154 | 0.070 | 0.122 | 0.129 | 0.058 | 0.069 | 0.089 | 0.183 | 0.075 | 0.070 | 0.076 | 0.112 |  | 0.357 | 0.437 | 0.304 | 0.376 | 0.423 | 0.420 | 0.484 | 0.453 | 0.461 |
| 14 | 0.120 | 0.088 | 0.195 | 0.150 | 0.093 | 0.106 | 0.152 | 0.271 | 0.129 | 0.198 | 0.082 | 0.205 | 0.211 |  | 0.591 | 0.443 | 0.492 | 0.544 | 0.553 | 0.573 | 0.526 | 0.610 |
| 15 | 0.363 | 0.323 | 0.295 | 0.398 | 0.313 | 0.239 | 0.197 | 0.442 | 0.439 | 0.253 | 0.292 | 0.414 | 0.222 | 0.386 |  | 0.194 | 0.213 | 0.122 | 0.204 | 0.243 | 0.313 | 0.263 |
| 16 | 0.229 | 0.179 | 0.176 | 0.249 | 0.266 | 0.174 | 0.170 | 0.350 | 0.316 | 0.188 | 0.258 | 0.337 | 0.213 | 0.329 | 0.067 |  | 0.127 | 0.102 | 0.093 | 0.247 | 0.295 | 0.269 |
| 17 | 0.262 | 0.199 | 0.181 | 0.298 | 0.189 | 0.155 | 0.205 | 0.294 | 0.353 | 0.236 | 0.187 | 0.269 | 0.229 | 0.252 | 0.031 | 0.054 |  | 0.060 | 0.101 | 0.257 | 0.315 | 0.300 |
| 18 | 0.295 | 0.247 | 0.200 | 0.329 | 0.249 | 0.177 | 0.163 | 0.339 | 0.368 | 0.245 | 0.255 | 0.320 | 0.238 | 0.351 | 0.019 | 0.025 | 0.010 |  | 0.045 | 0.192 | 0.266 | 0.227 |
| 19 | 0.371 | 0.294 | 0.248 | 0.408 | 0.318 | 0.240 | 0.196 | 0.423 | 0.434 | 0.334 | 0.302 | 0.399 | 0.263 | 0.404 | 0.015 | 0.027 | 0.017 | 0.002 |  | 0.267 | 0.352 | 0.291 |
| 20 | 0.347 | 0.351 | 0.252 | 0.419 | 0.317 | 0.262 | 0.230 | 0.347 | 0.479 | 0.301 | 0.372 | 0.378 | 0.308 | 0.474 | 0.051 | 0.077 | 0.060 | 0.026 | 0.066 |  | 0.175 | 0.192 |
| 21 | 0.268 | 0.273 | 0.209 | 0.415 | 0.236 | 0.256 | 0.176 | 0.280 | 0.361 | 0.227 | 0.290 | 0.280 | 0.278 | 0.345 | 0.082 | 0.118 | 0.092 | 0.050 | 0.098 | 0.026 |  | 0.272 |
| 22 | 0.279 | 0.287 | 0.223 | 0.342 | 0.268 | 0.207 | 0.163 | 0.308 | 0.426 | 0.380 | 0.317 | 0.322 | 0.236 | 0.403 | 0.036 | 0.091 | 0.056 | 0.047 | 0.054 | 0.019 | 0.049 |  |
