# Supplementary material for: Historical isolation and contemporary gene flow drive population diversity of the brown alga Sargassum thunbergii along the coast of China
Source: BMC Evol Biol. 2017 Dec 7;17:246. doi: 10.1186/s12862-017-1089-6 (PMC5721624; doi:10.1186/s12862-017-1089-6)
Supplement: Supplementary file 6 — F ST values between 22 Sargassum thunbergii populations based plastid rbc spacer. (DOCX 16 kb) [file 12862_2017_1089_MOESM6_ESM.docx]

**Additional file 6: Table S5:** *F*_ST_ values between 22 *Sargassum thunbergii* populations based plastid *rbc* spacer. Values inside box indicated *F*_ST_ between populations in North and South China. Statistically significant *F*_ST_ estimates are marked in bold (*p* < 0.05). Codes are the same as Table S1.

|  | 1 | 2 | 3 | 4 | 5 | 6 | 7 | 8 | 9 | 10 | 11 | 12 | 13 | 14 | 15 | 16 | 17 | 18 | 19 | 20 | 21 | 22 |
| --- | --- | --- | --- | --- | --- | --- | --- | --- | --- | --- | --- | --- | --- | --- | --- | --- | --- | --- | --- | --- | --- | --- |
| 1 | 0.000 |  |  |  |  |  |  |  |  |  |  |  |  |  |  |  |  |  |  |  |  |  |
| 2 | 0.004 | 0.000 |  |  |  |  |  |  |  |  |  |  |  |  |  |  |  |  |  |  |  |  |
| 3 | 0.012 | 0.000 | 0.000 |  |  |  |  |  |  |  |  |  |  |  |  |  |  |  |  |  |  |  |
| 4 | -0.003 | 0.000 | 0.000 | 0.000 |  |  |  |  |  |  |  |  |  |  |  |  |  |  |  |  |  |  |
| 5 | 0.011 | 0.000 | 0.000 | 0.000 | 0.000 |  |  |  |  |  |  |  |  |  |  |  |  |  |  |  |  |  |
| 6 | 0.011 | 0.000 | 0.000 | 0.000 | 0.000 | 0.000 |  |  |  |  |  |  |  |  |  |  |  |  |  |  |  |  |
| 7 | 0.006 | 0.000 | 0.000 | 0.000 | 0.000 | 0.000 | 0.000 |  |  |  |  |  |  |  |  |  |  |  |  |  |  |  |
| 8 | 0.007 | 0.000 | 0.000 | 0.000 | 0.000 | 0.000 | 0.000 | 0.000 |  |  |  |  |  |  |  |  |  |  |  |  |  |  |
| 9 | -0.003 | 0.000 | 0.000 | 0.000 | 0.000 | 0.000 | 0.000 | 0.000 | 0.000 |  |  |  |  |  |  |  |  |  |  |  |  |  |
| 10 | 0.006 | 0.000 | 0.000 | 0.000 | 0.000 | 0.000 | 0.000 | 0.000 | 0.000 | 0.000 |  |  |  |  |  |  |  |  |  |  |  |  |
| 11 | **0.089** | **0.172** | **0.194** | **0.152** | **0.190** | **0.190** | **0.176** | **0.179** | **0.152** | **0.176** | 0.000 |  |  |  |  |  |  |  |  |  |  |  |
| 12 | 0.012 | -0.010 | -0.005 | -0.015 | -0.006 | -0.006 | -0.009 | -0.008 | -0.015 | -0.009 | **0.177** | 0.000 |  |  |  |  |  |  |  |  |  |  |
| 13 | 0.010 | -0.008 | -0.003 | -0.014 | -0.004 | -0.004 | -0.007 | -0.007 | -0.014 | -0.007 | **0.169** | 0.000 | 0.000 |  |  |  |  |  |  |  |  |  |
| 14 | 0.015 | 0.036 | 0.045 | 0.026 | 0.044 | 0.044 | 0.037 | 0.039 | 0.026 | 0.037 | 0.020 | 0.035 | 0.033 | 0.000 |  |  |  |  |  |  |  |  |
|  |  |  |  |  |  |  |  |  |  |  |  |  |  |  |  |  |  |  |  |  |  |  |
| 15 | **0.770** | **0.865** | **0.876** | **0.854** | **0.875** | **0.875** | **0.867** | **0.869** | **0.854** | **0.867** | **0.644** | **0.861** | **0.856** | **0.780** | 0.000 |  |  |  |  |  |  |  |
| 16 | **0.904** | **1.000** | **1.000** | **1.000** | **1.000** | **1.000** | **1.000** | **1.000** | **1.000** | **1.000** | **0.835** | **0.973** | **0.972** | **0.930** | 0.092 | 0.000 |  |  |  |  |  |  |
| 17 | **0.902** | **1.000** | **1.000** | **1.000** | **1.000** | **1.000** | **1.000** | **1.000** | **1.000** | **1.000** | **0.833** | **0.973** | **0.972** | **0.929** | 0.090 | 0.000 | 0.000 |  |  |  |  |  |
| 18 | **0.448** | **0.526** | **0.551** | **0.502** | **0.547** | **0.547** | **0.530** | **0.534** | **0.502** | **0.530** | **0.251** | **0.556** | **0.546** | **0.419** | **0.130** | **0.317** | **0.313** | 0.000 |  |  |  |  |
| 19 | **0.902** | **1.000** | **1.000** | **1.000** | **1.000** | **1.000** | **1.000** | **1.000** | **1.000** | **1.000** | **0.833** | **0.973** | **0.972** | **0.929** | 0.090 | 0.000 | 0.000 | **0.313** | 0.000 |  |  |  |
| 20 | **0.727** | **0.841** | **0.855** | **0.826** | **0.853** | **0.853** | **0.843** | **0.846** | **0.826** | **0.843** | **0.578** | **0.839** | **0.832** | **0.739** | -0.031 | **0.152** | **0.149** | 0.074 | **0.149** | 0.000 |  |  |
| 21 | **0.904** | **1.000** | **1.000** | **1.000** | **1.000** | **1.000** | **1.000** | **1.000** | **1.000** | **1.000** | **0.835** | **0.973** | **0.972** | **0.930** | 0.092 | 0.000 | 0.000 | **0.317** | 0.000 | **0.152** | 0.000 |  |
| 22 | **0.891** | **1.000** | **1.000** | **1.000** | **1.000** | **1.000** | **1.000** | **1.000** | **1.000** | **1.000** | **0.813** | **0.970** | **0.969** | **0.921** | 0.075 | 0.000 | 0.000 | **0.287** | 0.000 | 0.127 | 0.000 | 0.000 |
