# Supplementary material for: Historical isolation and contemporary gene flow drive population diversity of the brown alga Sargassum thunbergii along the coast of China
Source: BMC Evol Biol. 2017 Dec 7;17:246. doi: 10.1186/s12862-017-1089-6 (PMC5721624; doi:10.1186/s12862-017-1089-6)
Supplement: Supplementary file 7 — Analysis of molecular variance (AMOVA) to partition genetic variance in the Northwest Pacific Sargassum thunbergii based on rbc spacer and SSR. (DOCX 14 kb) [file 12862_2017_1089_MOESM7_ESM.docx]

**Additional file 7: Table S6:** Analysis of molecular variance (AMOVA) to partition genetic variance in the Northwest Pacific *Sargassum thunbergii* based on *rbc* spacer and SSR.

| Markers | Source of variation | d.f. | %var | Fixation indices |
| --- | --- | --- | --- | --- |
| *rbc* spacer | Among clusters | 1 | 89.03 | Φ_CT_ = 0.890^***^ |
|  | Among populations within clusters | 20 | 1.70 | Φ_SC_ = 0.155^***^ |
|  | Within populations | 639 | 9.27 | Φ_ST_ = 0.907^***^ |
| SSR | Among clusters | 1 | 22.33 | Φ_CT_ = 0.223^***^ |
|  | Among populations within clusters | 20 | 12.29 | Φ_SC_ = 0.158^***^ |
|  | Within populations | 1078 | 65.38 | Φ_ST_ = 0.346^***^ |

^***^*P* < 0.0001
