# Supplementary material for: Historical isolation and contemporary gene flow drive population diversity of the brown alga Sargassum thunbergii along the coast of China
Source: BMC Evol Biol. 2017 Dec 7;17:246. doi: 10.1186/s12862-017-1089-6 (PMC5721624; doi:10.1186/s12862-017-1089-6)

**Additional file 8: Fig. S2:**Most likely number of genetic clusters using the Evanno method for *Sargassum thunbergii* individuals using 11 microsatellites from all the localities along the coast of China.


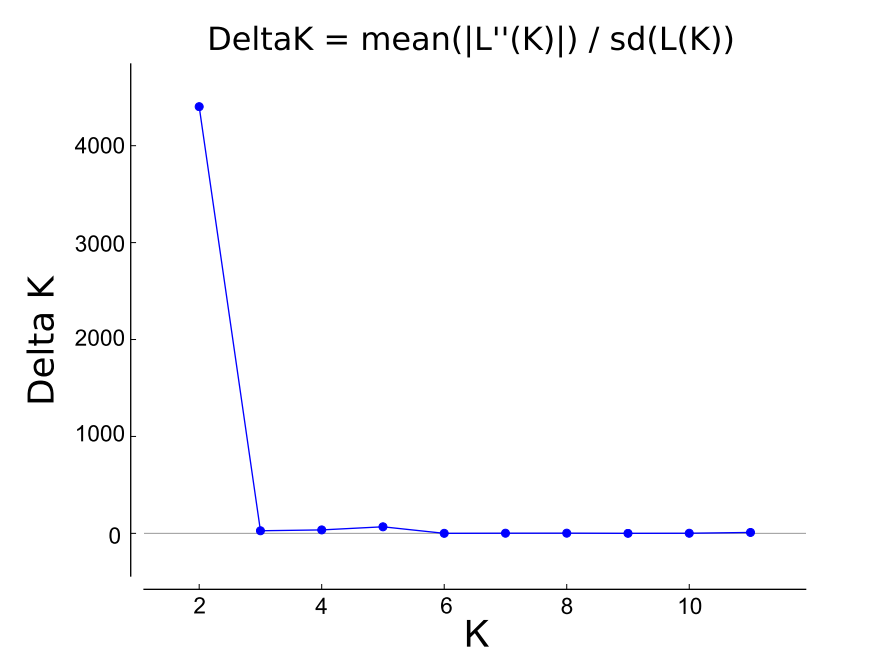

Supplement: Supplementary file 8 — Most likely number of genetic clusters using the Evanno method for Sargassum thunbergii individuals using 11 microsatellites from all the localities along the coast of China. (DOCX 51 kb) [file 12862_2017_1089_MOESM8_ESM.docx]
