# Supplementary material for: Historical isolation and contemporary gene flow drive population diversity of the brown alga Sargassum thunbergii along the coast of China
Source: BMC Evol Biol. 2017 Dec 7;17:246. doi: 10.1186/s12862-017-1089-6 (PMC5721624; doi:10.1186/s12862-017-1089-6)

**Additional file 9: Fig. S3:** UPGMA tree of 22 *Sargassum thunbergii* populatiosns based on microsatellites. Bootstrap > 50% is shown above the branches. POP1-14 are from the Yellow-bohai Sea (red); POP15-22 are from the East China Sea (Blue). Detailed locality information is shown in Table 1 and Fig. 2.


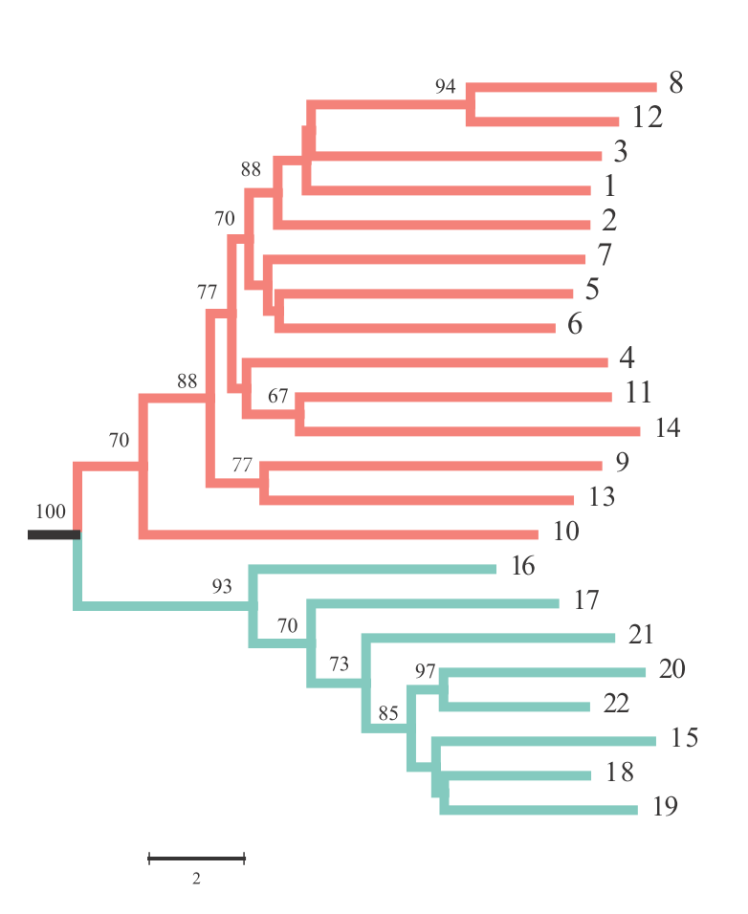

Supplement: Supplementary file 9 — UPGMA tree of 22 Sargassum thunbergii populations based on microsatellites. (DOCX 53 kb) [file 12862_2017_1089_MOESM9_ESM.docx]
